# Excellent clinical outcomes and retention in care for adults with HIV-associated Kaposi sarcoma treated with systemic chemotherapy and integrated antiretroviral therapy in rural Malawi

**DOI:** 10.7448/IAS.18.1.19929

**Published:** 2015-05-29

**Authors:** Michael E Herce, Noel Kalanga, Emily B Wroe, James W Keck, Felix Chingoli, Listern Tengatenga, Satish Gopal, Atupere Phiri, Bright Mailosi, Junior Bazile, Jason A Beste, Shekinah N Elmore, Jonathan T Crocker, Jonas Rigodon

**Affiliations:** 1 Department of Medicine, University of North Carolina School of Medicine, Chapel Hill, NC, USA; 2 Abwenzi Pa Za Umoyo, Neno, Malawi; 3 Partners In Health, Boston, MA, USA; 4 Department of Medicine, Brigham & Women's Hospital, Boston, MA, USA; 5 Department of Family Medicine, Tufts University School of Medicine, Boston, MA, USA; 6 Neno District Health Office, Malawi Ministry of Health, Neno, Malawi; 7 Division of Allergy & Infectious Diseases, Department of Medicine, University of Washington School of Medicine, Seattle, WA, USA; 8 Harvard Medical School, Boston, MA, USA; 9 Department of Medicine, Beth Israel Deaconess Hospital, Boston, MA, USA; 10 Haiti Assistance Program, American Red Cross, Washington, DC, USA

**Keywords:** Kaposi sarcoma, antiretroviral therapy, community health worker, Malawi, paclitaxel, bleomycin, vincristine, psychosocial support

## Abstract

**Introduction:**

HIV-associated Kaposi sarcoma (HIV-KS) is the most common cancer in Malawi. In 2008, the non-governmental organization, Partners In Health, and the Ministry of Health established the Neno Kaposi Sarcoma Clinic (NKSC) to treat HIV-KS in rural Neno district. We aimed to evaluate 12-month clinical outcomes and retention in care for HIV-KS patients in the NKSC, and to describe our implementation model, which featured protocol-guided chemotherapy, integrated antiretroviral therapy (ART) and psychosocial support delivered by community health workers.

**Methods:**

We conducted a retrospective cohort study using routine clinical data from 114 adult HIV-KS patients who received ART and ≥1 chemotherapy cycle in the NKSC between March 2008 and February 2012.

**Results:**

At enrolment 97% of patients (*n*/*N*=103/106) had advanced HIV-KS (stage T1). Most patients were male (*n*/*N*=85/114, 75%) with median age 36 years (interquartile range, IQR: 29–42). Patients started ART a median of 77 days prior to chemotherapy (IQR: 36–252), with 97% (*n*/*N*=105/108) receiving nevirapine/lamivudine/stavudine. Following standardized protocols, we treated 20 patients (18%) with first-line paclitaxel and 94 patients (82%) with bleomycin plus vincristine (BV). Of the 94 BV patients, 24 (26%) failed to respond to BV requiring change to second-line paclitaxel. A Division of AIDS grade 3/4 adverse event occurred in 29% of patients (*n*/*N*=30/102). Neutropenia was the most common grade 3/4 event (*n*/*N*=17/102, 17%). Twelve months after chemotherapy initiation, 83% of patients (95% CI: 74–89%) were alive, including 88 (77%) retained in care. Overall survival (OS) at 12 months did not differ by initial chemotherapy regimen (*p*=0.6). Among patients with T1 disease, low body mass index (BMI) (adjusted hazard ratio, aHR=4.10, 95% CI: 1.06–15.89) and 1 g/dL decrease in baseline haemoglobin (aHR=1.52, 95% CI: 1.03–2.25) were associated with increased death or loss to follow-up at 12 months.

**Conclusions:**

The NKSC model resulted in infrequent adverse events, low loss to follow-up and excellent OS. Our results suggest it is safe, effective and feasible to provide standard-of-care chemotherapy regimens from the developed world, integrated with ART, to treat HIV-KS in rural Malawi. Baseline BMI and haemoglobin may represent important patient characteristics associated with HIV-KS survival in rural sub-Saharan Africa.

## Introduction

Kaposi sarcoma (KS) is a major cause of morbidity and mortality in sub-Saharan Africa (SSA), resulting in 25,000 deaths each year [1]. In Malawi, KS is the most common cancer, accounting for 35% of approximately 8,000 new cancer cases registered annually [2].

Although dramatic reductions in KS incidence and mortality have been observed in Western countries in the antiretroviral therapy (ART) era, similar reductions have yet to be achieved in SSA where prevalence of HIV-1 and KS-associated herpes virus (KSHV) is high and where ART access is more limited [3–9]. As a result, poor 12-month overall survival (OS) for HIV-KS has frequently been reported in SSA, especially for patients not receiving ART [10–12]. Even with recent increasing ART availability, 12-month OS is lower for HIV-infected patients with KS than those without KS. Among patients receiving ART in Malawi, only 53% of patients with HIV-KS were alive at 12 months compared to 66% without HIV-KS [13].

Chemotherapy combined with ART improves disease response and survival in advanced HIV-KS [14–16]. Several agents are effective for HIV-KS, including vincristine, bleomycin, doxorubicin and etoposide [17–19]. Newer drugs, including liposomal doxorubicin and paclitaxel, have superior efficacy and tolerability compared to older agents. Monotherapy with either drug is the standard of care for first- or second-line HIV-KS treatment in developed countries [16, 20–23].

In SSA, limited data exist describing the safety, efficacy and feasibility of HIV-KS chemotherapy regimens, and characterizing promising models of HIV-KS treatment delivery [12, 24, 25]. Only one clinical trial from SSA—the KAART trial from Durban, South Africa—has been reported in the ART era. This study demonstrated that ART plus chemotherapy (doxorubicin, bleomycin, vincristine; ABV) achieved superior disease response and similar 12-month OS compared to ART alone [26]. The implications of these results are unclear for HIV-KS treatment provided outside urban centres, including rural areas where nearly two-thirds of people in SSA live [27].

To address HIV-KS in rural Malawi, the non-governmental organization (NGO), Partners In Health (PIH), and the Malawi Ministry of Health (MoH) jointly established the Neno Kaposi Sarcoma Clinic (NKSC) in 2008. We report here clinical outcomes and the implementation model for the NKSC program.

## Methods

### Study site

NKSC is held weekly at Neno District Hospital in Neno district, a rural district in southern Malawi with 130,612 people [28]. MoH, with PIH support, provides free HIV testing and counselling, ART and other essential health services at all Neno health facilities.

### Implementation model

Patients with suspected HIV-KS are referred to NKSC from Neno hospitals and clinics. Patients may also be referred from outside Neno district. At the initial visit, all patients undergo a thorough evaluation. Most HIV-KS is diagnosed clinically, as recommended by national HIV guidelines due to limited diagnostic pathology availability in Malawi [29–31]. Biopsies are performed to confirm HIV-KS when the clinical diagnosis is uncertain, or at the discretion of the treating clinician. Trained clinical officers, working with a PIH physician, perform comprehensive clinical assessments using a standardized form (Supplementary file 1); administer protocol-guided chemotherapy following standardized protocols (Supplementary file 2); and complete a total body pictorial chart to document KS lesion size, location, and associated oedema and/or ulceration (Supplementary file 3). HIV-KS is staged using AIDS Clinical Trials Group (ACTG) criteria modified for settings without routine CD4 count monitoring [25, 30, 32, 33]. Investigations for other KSHV-associated conditions (e.g. multicentric Castleman's disease) are not routinely performed due to limited laboratory and pathology services in our setting [31].

All HIV-KS patients receive ART, adherence counselling, analgesia and symptom management, and longitudinal clinical monitoring [30]. Patients with T0 limited disease responding to ART may be managed with ART alone, following national guidelines [30]. Qualifying HIV-KS patients without a contraindication to systemic chemotherapy—i.e. absolute neutrophil count <1,000/mm^3^, haemoglobin <7.0 g/dL, platelets <75,000/mm^3^, or other Division of AIDS (DAIDS) grade 3/4 abnormality—receive bleomycin plus vincristine (BV) or paclitaxel in addition to ART. The initial chemotherapy regimen is based on extent of disease. Once-weekly outpatient BV is typically provided to patients with widespread skin involvement (e.g. >20 lesions) with or without associated oedema [20]. Patients with T1 disease and any of the following are eligible for first-line paclitaxel: ulcerative or fungating KS; symptomatic visceral involvement; scrotal or bilateral lower extremity oedema, or oedema interfering with ambulation; or nodular oral KS extending beyond the palate. For BV, bleomycin is given as a 15 IU intramuscular (IM) injection, and vincristine as a slow 2 mg intravenous (IV) bolus with pre- and post-bolus normal saline flush to minimize risk of extravasation. During most of the evaluation period (i.e. March 2008 to January 2012), a 1 mg vincristine dose was used because of drug cost and availability. We intentionally chose a once-weekly BV schedule to promote close follow-up for an ill patient population and to account for the lower doses of vincristine and bleomycin used in the NKSC compared with more traditional biweekly regimens [17, 23]. We selected IM and IV bolus routes of administration to maximize the operational feasibility of BV administration in a busy clinic with limited staffing.

Patients receiving BV with new or unchanged lesions after ≥10 cycles, or otherwise with clinically assessed disease progression are eligible for second-line paclitaxel. Patients prescribed paclitaxel receive pre-hydration, premedication (with IV dexamethasone, IV promethazine and oral cimetidine) and chemotherapy every three weeks up to eight cycles. Paclitaxel was initially dosed at 100 mg/m^2^ due to concerns about myelosuppression. Following an interim program evaluation establishing paclitaxel safety at this dose, the protocol was modified in March 2012 to incorporate a dose of 135 mg/m^2^. After paclitaxel administration, patients receive a five-day ciprofloxacin prescription and instructions to start the antibiotic in case of fever, chills, or malaise suggesting possible febrile neutropenia. We provide paclitaxel-treated patients with an antibiotic prescription because of paclitaxel's known myelosuppressive effects, the extended three-week interval between visits and the distances required to reach medical attention in our rural setting [20].

Before each cycle, patients undergo an interval history, physical examination and laboratory testing for serum creatinine, liver function and complete blood count. In cases of a severe laboratory abnormality or other adverse event, chemotherapy is postposed until the laboratory parameter normalizes or the event resolves. Before BV cycles 1, 10 and 20, and paclitaxel cycles 1 and 8, a comprehensive clinical assessment is conducted that includes counting mucocutaneous KS lesions and reassessment of visceral disease as clinically indicated using chest x-ray, rectal exam and stool occult blood testing. All patients undergo comprehensive follow-up assessment four weeks after their last chemotherapy. We refer patients to a medical oncologist in Blantyre if there is no response after eight cycles of paclitaxel given the likelihood of an adverse event and/or disease progression with continued paclitaxel, or if chemotherapy dose modification is required after an adverse event.

Patients with ambulation-limiting oedema, ulcerations, or dyspnoea receive transportation reimbursement (approximately $6). Paid PIH community health workers (CHWs) provide each patient from Neno district with psychosocial support through routine home visits [34]. CHWs promote ART and chemotherapy adherence, and identify interim illnesses requiring medical attention. When such events are identified, CHWs escort patients to the nearest health facility.

### Study design and population

**Figure 1:**
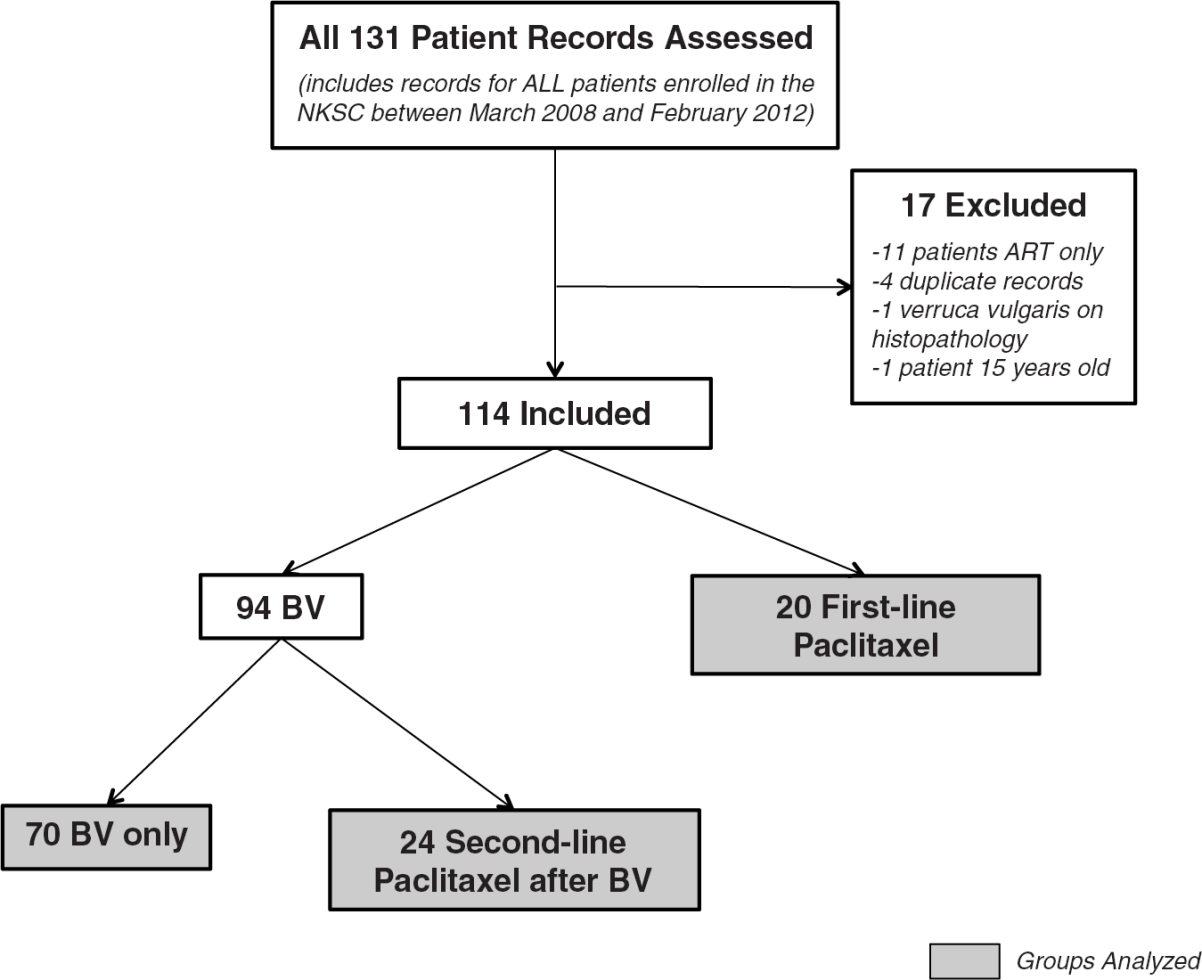
Patient flow chart. Depicts study inclusion and exclusion criteria as well as chemotherapy regimens prescribed (NKSC: Neno Kaposi Sarcoma Clinic; BV: bleomycin plus vincristine; ART: antiretroviral therapy).

We conducted a retrospective cohort study of adult NKSC patients receiving HIV-KS services between March 2008 and February 2012. Inclusion criteria were: HIV infection; ART receipt; KS diagnosed clinically or by histopathology; no prior combination chemotherapy; and receipt of ≥1 cycle of NKSC protocol-guided chemotherapy. We excluded pregnant women and children <18 years old. We reviewed 131 patient records, of which 114 met inclusion criteria (Figure 1).

### Data collection

Data were collected during routine clinical care. We abstracted the following data from patient paper charts and an institutional electronic medical record (EMR): sociodemographics, HIV diagnosis date, opportunistic infection history, ART start date and regimen, KS diagnosis date, KS burden, ACTG stage, CD4 counts, laboratory data, chemotherapy doses, cycles completed and 12-month OS [35]. We defined survival outcomes as death, loss to follow-up (LTFU—no clinic visit within three months prior to April 30, 2013), transfer out, and alive and retained in care. We censored all survival outcomes on April 30, 2013. We assessed vital status from patient charts, the EMR, ART registers and verbal autopsy with next-of-kin. We censored patients at the date of transfer or their last NKSC encounter. We reviewed all charts and EMR entries to assess toxicity, which was graded retrospectively by the authors according to DAIDS criteria [36].

### Data analyses

We present summary statistics for baseline variables and clinical outcomes, including frequencies and percentages for categorical variables, and means or medians and measures of dispersion for continuous variables. We analysed relationships between baseline variables and clinical outcomes using Chi-square and Fisher's exact tests. We estimated survival, controlling for censoring, using the Kaplan-Meier method with follow-up time measured from chemotherapy initiation. We compared survivor functions using the Wilcoxon test and test for trend, as appropriate. We used Cox-proportional hazard modelling to estimate exploratory associations between baseline covariates and our composite outcome of mortality or LTFU at 12 months. We selected covariates for our Cox model based on clinical plausibility. We combined death and LTFU in our Cox models to more conservatively estimate associations between covariates of interest and survival, given high mortality among patients experiencing LTFU in resource-limited settings [37]. We considered two-sided *p*-values ≤0.05 statistically significant. All statistical analyses were performed using STATA version 12.1 (College Station, TX, USA).

### Ethics statement

The study was approved by the National Health Sciences Research Committee of Malawi, the Partners Human Research Committee, USA, and the Institutional Review Board of the University of North Carolina, USA, without requiring patient consent given the retrospective use of de-identified routinely collected clinical data.

## Results

### Patient characteristics

Of 114 patients, 85 were male (75%) with median age 36 years (interquartile range, IQR: 29–42) (Table 1). Most patients (*n*/*N*=87/107, 81%) resided in Neno district; 19% (*n*/*N*=20/107) presented from other districts. Median baseline body mass index (BMI) was 22 kg/m^2^ (IQR: 20–24). Most patients (*n*/*N*=78/109, 72%) had KS diagnosed clinically; 31 were diagnosed (28%) by histopathology. Mean haemoglobin was 10.6 g/dL (standard error: 0.2), and 30% (*n*/*N*=31/103) had a haemoglobin <9.5 g/dL [38]. Median white blood cell (WBC) count was 5.4×10^3^ cells/mm^3^ (IQR: 4.2×10^3^–7.2×10^3^) [38]. Thirty-one patients (27%) had a documented baseline CD4 count, among whom median CD4 count was 269 cells/mm^3^ (IQR: 171–454). According to Malawi guidelines, CD4 count testing is not required to initiate ART in patients with WHO stage III/IV disease [30].

**Table 1:** Baseline patient characteristics

Characteristics	Full cohort, *N*=114^a^ *n* (%)
Age (years)	
≥50	14 (12)
36–49	43 (38)
≤35	57 (50)
Sex	
Male	85 (75)
Female	29 (25)
Body mass index (BMI)	
BMI ≥19 kg/m^2^	97 (89)
BMI<19 kg/m^2^	12 (11)
White blood cell (WBC) count	
WBC>9100 cells/mm^3^	14 (13)
3100 cells/mm^3^≤WBC≤9100 cells/mm^3^	85 (82)
WBC<3100 cells/mm^3^	5 (5)
Haemoglobin (Hgb)	
Hgb ≥9.5 g/dL	72 (70)
Hgb<9.5 g/dL	31 (30)
ART status at enrolment	
Already on ART at enrolment	104 (96)
Started ART at enrolment	4 (4)
Duration on ART prior to first chemotherapy dose (days)	
ART duration>180	33 (32)
30<ART duration ≤180	45 (44)
ART duration ≤30	24 (24)
Indication for ART	
WHO stage IV condition – KS	65 (64)
WHO stage III condition (excluding TB)	16 (16)
CD4 count<250 (cells/mm^3^)	11 (11)
WHO stage III – TB	6 (6)
Other WHO stage IV condition	3 (3)
ART regimen at enrolment	
NVP/3TC/d4T	105 (97)
NVP/3TC/AZT	2 (2)
LPV/r+AZT/3TC/TDF	1 (1)
KS T stage (ACTG) at enrolment	
T1^b^	103 (97)
T0	3 (3)

a Values that do not sum to 114 reflect missing data

b T1: oedema or ulceration, extensive oral mucosa KS, or visceral KS; indicates ACTG “Poor Risk” disease.

ART: antiretroviral therapy; NVP/3TC/d4T: nevirapine/lamivudine/stavudine; NVP/3TC/AZT: nevirapine/lamivudine/zidovudine; LPV/r+AZT/3TC/TDF: lopinavir/ritonavir+zidovudine/lamivudine/tenofovir; T stage: tumour extent; ACTG: AIDS Clinical Trials Group staging.

Patients started ART a median of 77 days prior to chemotherapy (IQR: 36–252), with 24% (*n*/*N*=24/102) on ART ≤30 days at enrolment (Table 1). As per Malawi guidelines during the study period, 97% of patients (*n*/*N*=105/108) received nevirapine/lamivudine/stavudine. Most (*n*/*N*=104/108, 96%) initiated ART prior to NKSC enrolment. Sixty-five patients (*n*/*N*=65/101, 64%) started ART for HIV-KS, a WHO stage IV condition. T1 disease was present in 97% of patients (*n*/*N*=103/106), and 75% (*n*/*N*=72/96) had>20 documented KS lesions at baseline.

### Chemotherapy

Twenty patients (18%) with T1 disease received first-line paclitaxel and completed a median of eight cycles (IQR: 8–8) over a median 175 days (IQR: 137–217). The median cumulative dose of first-line paclitaxel was 804 mg/m^2^ (IQR: 720–859). No patient switched to BV from first-line paclitaxel. Ninety-four patients (82%) started treatment with BV. Of these, 70 patients (74%) received BV alone (median 20 cycles, IQR: 12–20; median duration 153 days, IQR: 98–175). Median cumulative doses of bleomycin and vincristine were 300 IU (IQR: 180–300) and 20 mg (IQR: 14–20), respectively. Twenty-four patients (26%) received BV (median 19 cycles, IQR: 10–20; median cumulative bleomycin dose 280 IU, IQR: 150–300; median cumulative vincristine dose 19 mg, IQR: 10–20), followed by second-line paclitaxel (median seven cycles, IQR: 6–8; median cumulative dose 703 mg/m^2^, IQR: 555–797).

### Overall survival

**Figure 2:**
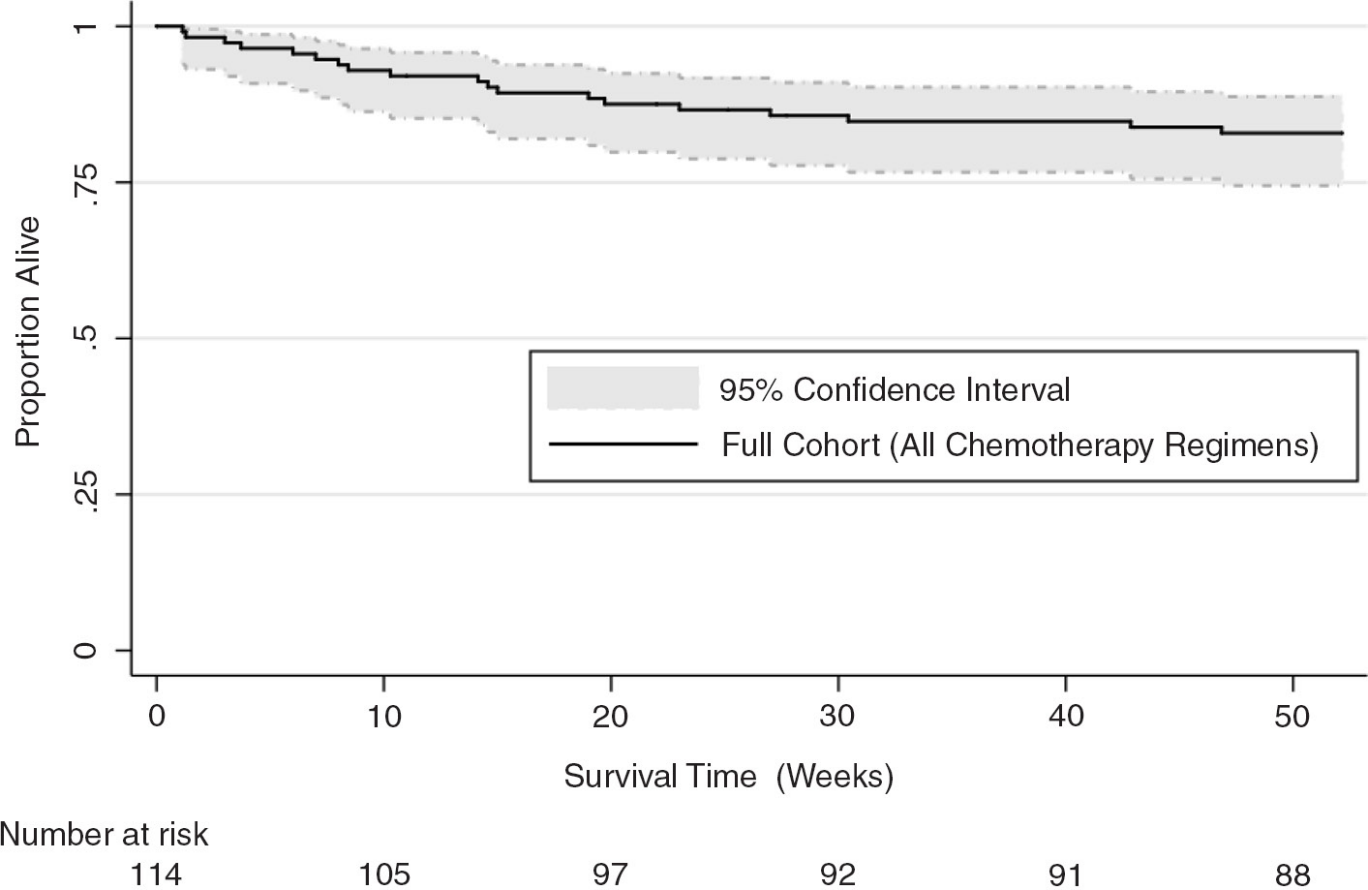
Twelve-month overall survival for the Neno Kaposi Sarcoma Clinic full cohort (*N*=114).

Median follow-up was 86 weeks (IQR: 55–169). Twelve months after chemotherapy initiation, 88 of 114 patients (77%) were alive and retained in care, 19 patients (17%) had died, six patients (5%) were lost to follow-up and one patient (1%) had transferred out. Estimated 12-month OS for the full cohort was 83% (95% confidence interval, CI: 74–89%) (Figure 2). Median time to death was 99 days (IQR: 42–161). For patients receiving first- or second-line paclitaxel, 35 of 44 patients (80%) were alive and retained in care at 12 months, seven patients (16%) had died, one patient was lost to follow-up (2%) and one patient had transferred out (2%). Estimated 12-month OS for this group was 84% (95% CI: 69–92%).

**Figure 3:**
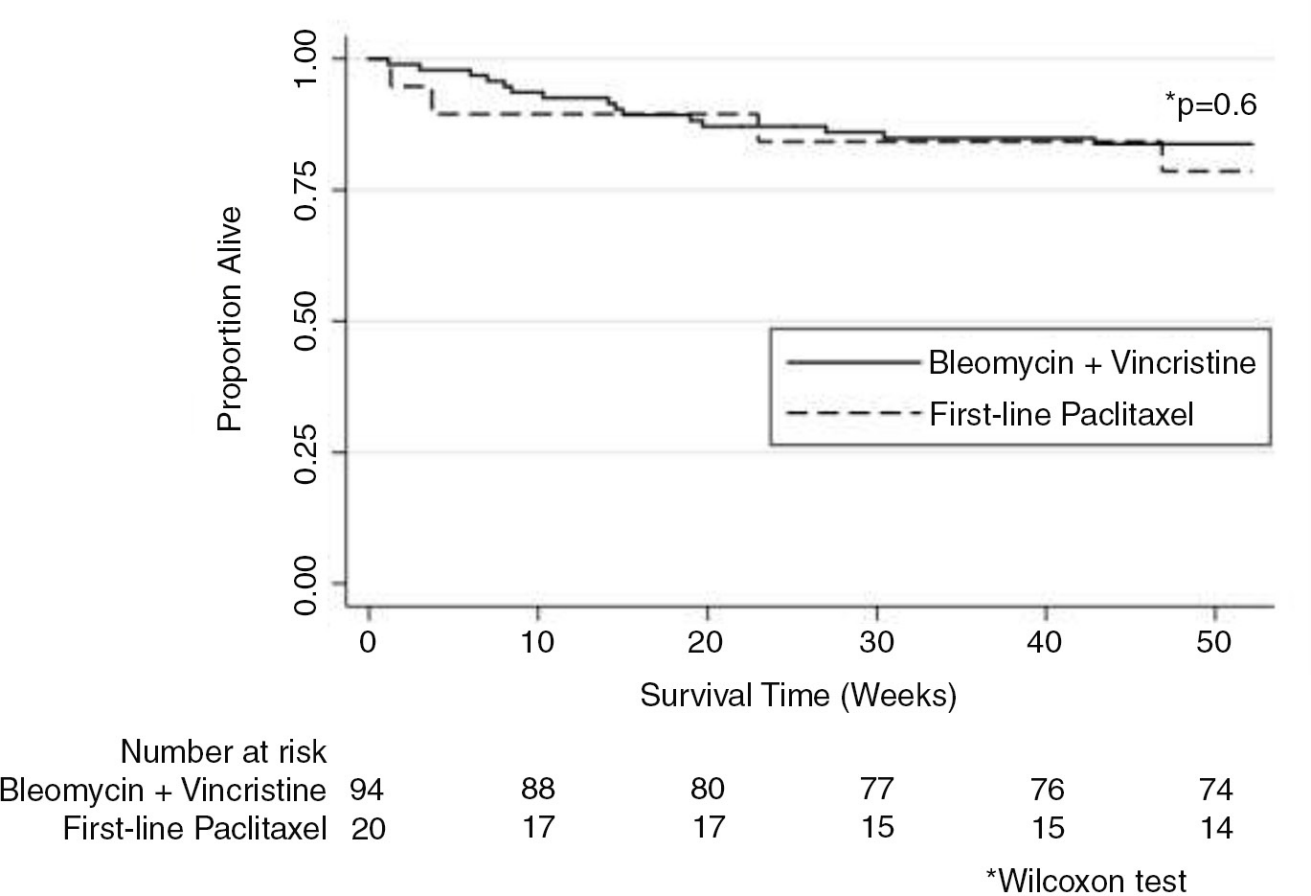
Kaplan-Meier survival estimates by initial chemotherapy regimen. Twelve-month overall survival stratified by initial chemotherapy regimen (bleomycin plus vincristine or first-line paclitaxel; *N*=114).

**Figure 4:**
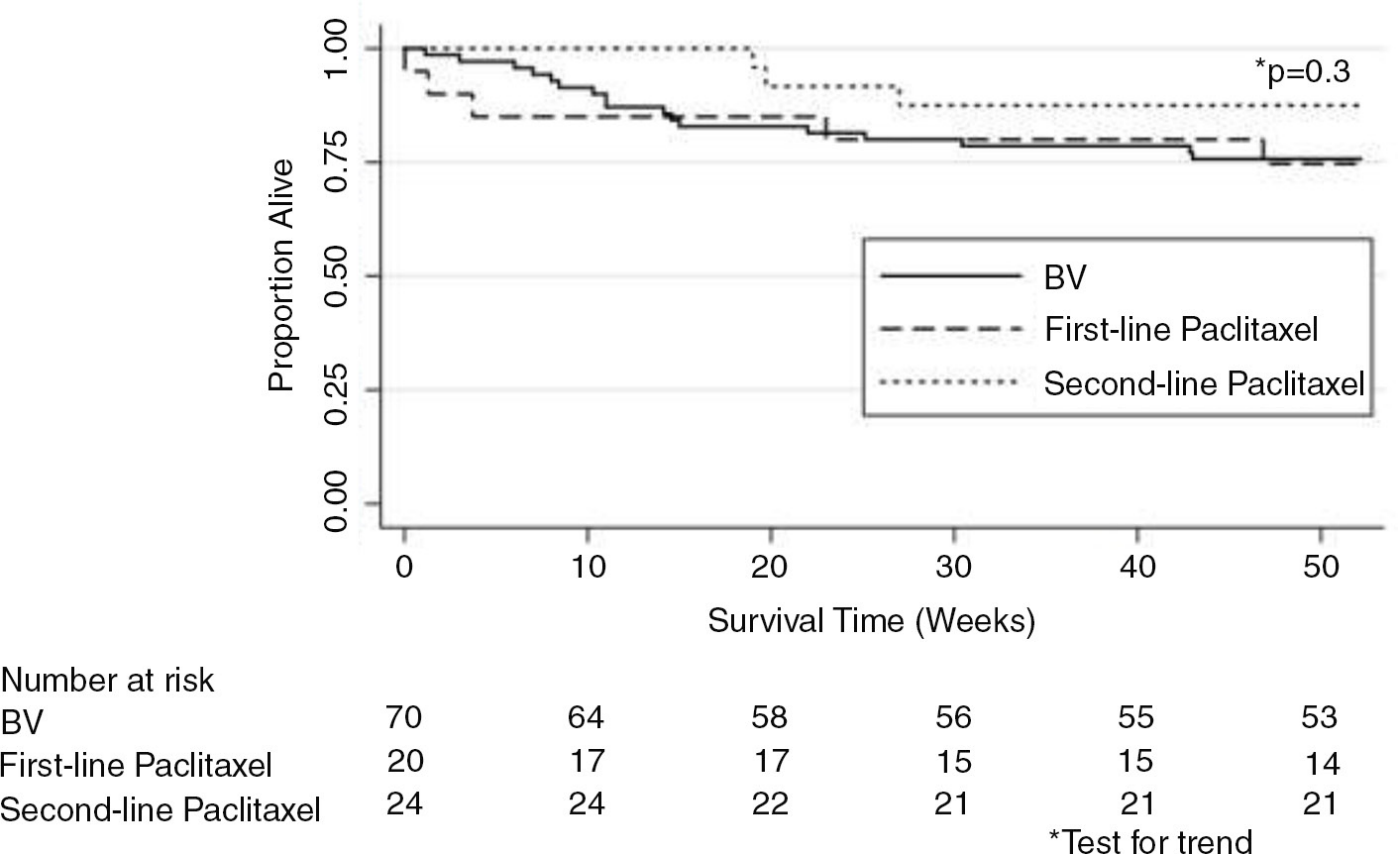
Kaplan-Meier survival estimates by final chemotherapy regimen. Twelve-month overall survival stratified by final chemotherapy regimen (bleomycin plus vincristine, BV, first-line paclitaxel, or second-line paclitaxel; *N*=114).

Twelve-month OS did not differ significantly by initial chemotherapy regimen prescribed (*p*=0.6) (Figure 3). Patients who failed BV as their initial regimen and required second-line paclitaxel had 12-month OS that was not significantly different from patients treated with BV or first-line paclitaxel alone (*p*=0.3) (Figure 4). We also did not observe any differences in OS based on initial (*p*=0.8) or final chemotherapy regimen (*p*=0.5) after restricting our analysis to patients with T_1_ disease. Malawi lacks death certification and, therefore, cause of death could not be reliably ascertained.

In unadjusted analyses restricted to patients with T1 disease, BMI <19 kg/m^2^ was significantly associated with death or LTFU at 12 months (unadjusted hazard ratio, HR=3.91, 95% CI: 1.51–10.11) (Table 2). Every 1 g/dL decrease in baseline haemoglobin was also significantly associated with death or LTFU at 12 months (unadjusted HR=1.27; 95% CI: 1.01–1.61). In adjusted analyses, low BMI (adjusted hazard ratio, aHR=4.10; 95% CI: 1.06–15.89) and 1 g/dL decrease in baseline haemoglobin (aHR=1.52, 95% CI: 1.03–2.25) remained significantly associated with death or LTFU at 12 months. Sex, age, chemotherapy regimen, adverse event history and ART duration at chemotherapy initiation were not significantly associated with the composite outcome in unadjusted or adjusted analyses.

**Table 2:** Exploratory analysis of characteristics associated with mortality or loss to follow-up at 12 months for patients with documented T1^a^ HIV-associated Kaposi sarcoma (*N*=103) treated in the Neno Kaposi Sarcoma Clinic (March 2008 to February 2012)

		Unadjusted		Adjusted^b^	
			
Characteristic		HR	95% CI	HR	95% CI
Age at enrolment	≥50 years	1.05	(0.31, 3.55)	1.68	(0.32, 8.76)
	<50 years	1.00			
Sex	Female	0.44	(0.13, 1.48)	0.30	(0.06, 1.52)
	Male	1.00			
ART duration at time of first chemotherapy	1 week increase in ART duration	0.97	(0.93, 1.01)	0.98	(0.93, 1.03)
Baseline body mass index (BMI)	<19 kg/m^2^	3.91^c^	(1.51, 10.11)	4.10^c^	(1.06, 15.89)
	≥19 kg/m^2^	1.00			
Baseline haemoglobin (g/dL)	1 g/dL decrease	1.27^c^	(1.01, 1.61)	1.52^c^	(1.03, 2.25)
Chemotherapy regimen	Second-line paclitaxel	0.44	(0.13, 1.51)	0.49	(0.10, 2.44)
	First-line paclitaxel	0.94	(0.31, 2.82)	0.71	(0.08, 6.41)
	Bleomycin plus vincristine	1.00			
Grade 3/4 adverse event on treatment	Yes	1.63	(0.66, 4.05)	1.05	(0.31, 3.54)
	No	1.00			

a T1: patients presenting with Kaposi sarcoma-associated oedema or ulceration, extensive oral mucosa KS, or visceral involvement

b HR estimated by Cox-proportional hazard modelling, adjusting for remaining variables presented in the table

c statistically significant association with death or loss to follow-up at 12 months (p≤0.05).

HR: hazard ratio; ART: antiretroviral therapy.

### Adverse events

Thirty patients (*n*/*N*=30/102, 29%) with complete laboratory monitoring had one or more documented DAIDS grade 3/4 event(s). Six patients (*n*/*N*=6/102, 6%) experienced >1 event. Sixteen patients (23%) receiving BV, four patients (20%) receiving first-line paclitaxel and 10 (42%) receiving second-line paclitaxel had ≥1 grade 3/4 event (Table 3). We observed a statistically significant association between final chemotherapy regimen and the proportion of patients experiencing ≥1 grade 3/4 event (*p*=0.04). More patients who received second-line paclitaxel experienced ≥1 grade 3/4 event compared to patients treated with BV alone (*p*=0.01) or first-line paclitaxel (*p*=0.06). Neutropenia was the most common grade 3/4 event (Table 3).

**Table 3:** DAIDS Grade 3/4 adverse events^a^: highest-grade event(s) per patient organized by class and final chemotherapy regimen^b^

Class	Event	Bleomycin plus vincristine (*N*=70)	First-line paclitaxel (*N*=20)	Second-line paclitaxel^c^ (*N*=24)	*p*
Haematologic (*n*)					
	Neutropenia	9	3^d^	2	
	Anaemia	4	0	2	
	Thrombocytopenia	0	1	1	
	Sub-total	13	4	5	
Hepatic (*n*)					
	ALT/AST elevation	1	0	0	
	Sub-total	1	0	0	
Cardiac (*n*)					
	Symptomatic hypotension requiring IV fluids	0	0	1^e^	
	Sub-total	0	0	1	
Multiple events (*n*)					
	Recurrent neutropenia	0	0	3	
	Neutropenia & anaemia	1	0	0	
	Neutropenia, anaemia, & ALT/AST elevation	1^f^	0	0	
	Neutropenia, thrombocytopenia, & ALT/AST elevation	0	0	1^g^	
	Sub-total	2	0	4	
Total *n* (%)		16 (23%)	4 (20%)	10 (42%)	0.04^h^

a DAIDS, Division of AIDS table for grading the severity of adult and paediatric adverse events version 1.0, December, 2004; Clarification August 2009 (www.rsc.tech-res.com/Document/safetyandpharmacovigilance/Table_for_Grading_Severity_of_Adult_Pediatric_Adverse_Events.pdf)

b The table lists the highest-grade, mutually exclusive grade 3/4 event or combination of events (i.e. “multiple events”) experienced by each patient, organized by final chemotherapy regimen and adverse event class. All adverse events were at least *possibly* related to chemotherapy based on U.S. National Cancer Institute nomenclature, unless otherwise indicated (www.ctep.cancer.gov/protocolDevelopment/electronic_applications/docs/aeguidelines.pdf)

c includes any DAIDS grade 3/4 event occurring from BV initiation through the last cycle of second-line paclitaxel

d includes one confirmed episode of fever and neutropenia that resolved with antibiotic treatment

e definitely related to second-line paclitaxel (based on U.S. National Cancer Institute nomenclature), as the event occurred during paclitaxel infusion and resolved with stopping paclitaxel

f patient developed grade 3 ALT elevation followed by grade 3 anaemia and grade 4 neutropenia

g patient developed grade 3 AST elevation and grade 3 neutropenia while receiving BV. Patient also developed grade 3 thrombocytopenia during treatment with second-line paclitaxel

h *p* value is for the overall association between final chemotherapy regimen and grade 3 or 4 severe adverse event(s). A significantly greater proportion of patients who received second-line paclitaxel experienced ≥1 grade 3 or 4 adverse event compared to patients who received bleomycin plus vincristine only (*p*=0.01). A non-significantly greater proportion of patients who received second-line paclitaxel experienced ≥1 grade 3 or 4 adverse event compared to patients who received first-line paclitaxel (*p*=0.06).

ALT (SGPT): alanine aminotransferase; AST (SGOT): aspartate aminotransferase; IV: intravenous.

## Discussion

Our results demonstrate that it is safe, effective and feasible to provide paclitaxel or combination BV integrated with ART to treat HIV-KS in rural Malawi. Our program provided comprehensive HIV-KS treatment using an implementation model featuring task shifting, community-level psychosocial support and infrastructure leveraged from a government–NGO partnership. This approach resulted in clinical outcomes comparable to those observed in trials from urban SSA referral centres and cohort studies from developed countries [26, 39].

Estimated one-year OS in our cohort was 83%. This compares favourably with prior reports from SSA, including one-year OS of 30 to 40% in Zimbabwe and Kenya prior to ART scale-up, 53% in Malawi for a cohort receiving ART alone and 77% for patients treated with ART plus ABV in the KAART trial [10, 11, 13, 26]. Although adding chemotherapy to ART did not improve OS compared to ART alone in KAART, observational data suggest that chemotherapy integrated with ART may reduce HIV-KS mortality in SSA [12, 26, 40].

Evidence also suggests that treating HIV-KS with integrated chemotherapy and ART improves disease response in SSA [26]. Due to health worker shortages and high clinical volume in the NKSC, we could not obtain detailed documentation of disease response for most patients using ACTG criteria. In our busy routine care setting, there was a tendency for clinical staff to provide more detailed response assessment documentation for patients with stable or progressive disease to justify chemotherapy switch or oncologist referral than to document the extent of response for patients responding to treatment. Because of these difficulties with response assessment documentation, we focused our analyses on OS. Our experience suggests that novel systems are needed to more efficiently capture HIV-KS disease response under routine clinical conditions in SSA.

In Malawi, vincristine plus ART remains recommended first-line treatment for T1 disease despite limited efficacy, owing to its low cost and favourable toxicity profile [30, 41]. Concerns about cost and toxicity, as well as perceived challenges of delivering chemotherapy in resource-limited settings, have been cited as reasons not to introduce newer, more effective chemotherapy regimens for HIV-KS in SSA. However, these concerns are not supported by our experience or existing data [24, 25, 42]. In the KAART trial, patients treated with ABV plus ART experienced no greater risk of severe adverse event, inadequate HIV control, or poor treatment adherence than patients receiving ART alone [24, 26]. In our program, estimated costs for BV ($335 USD) and paclitaxel ($580 USD) treatment were comparable to annual costs for ART, and less than reported cost-effectiveness thresholds for SSA [43, 44].

We chose combination BV and paclitaxel for their relative affordability, favourable safety profiles and superior efficacy compared to vincristine monotherapy [17, 21, 45, 46]. The superior tumour response and comparable safety of BV compared to vincristine has recently been demonstrated in Lilongwe, Malawi [46]. For paclitaxel, we were persuaded by efficacy data establishing paclitaxel as a standard-of-care agent for HIV-KS in developed countries [21, 22]. Despite this evidence, however, reports of paclitaxel's use in SSA are scarce, possibly because paclitaxel poses unique issues for HIV-KS management, including the need to pre-medicate patients with corticosteroids [47] and monitor for drug–drug interactions in patients receiving non-nucleoside reverse transcriptase inhibitors [20]. We believed premedication and drug-interaction issues for paclitaxel could be successfully managed even in our rural Malawi context, and observed relatively few grade 3/4 events and excellent OS for paclitaxel-treated patients.

Key interventions to ensure safety in our setting included: standardized premedication; CHW support to identify interim illnesses and refer sick patients urgently to care; and antibiotic prescription after chemotherapy for early home-based treatment of possible febrile neutropenia [20]. With these interventions, approximately one-third of NKSC patients experienced a grade 3/4 event, and treatment-related toxicity was associated with the intensity and duration of chemotherapy, as expected. Adverse event frequency may have been underestimated due to limited human resources and laboratory capacity for toxicity surveillance in our setting. These limitations precluded routine HIV viral load and serum chemistry monitoring and may have limited detection of vincristine-related neurotoxicity, renal injury and episodes of KS-related immune reconstitution inflammatory syndrome.

Limited laboratory capacity also made it difficult to pathologically confirm KS in all patients. Accordingly, we diagnosed KS mostly on clinical grounds, reflecting the standard of care in Malawi [30]. While KS misdiagnosis may occur under such circumstances, we purposefully biopsied patients with atypical KS lesions in whom alternative, non-KS diagnoses were the most likely [48, 49]. This limitation notwithstanding, more NKSC patients received a pathologically confirmed diagnosis (28%) than previously reported in South Africa (3%) or Blantyre, Malawi (17%) [12, 50]. Other study limitations included our use of routinely collected clinical information, which resulted in missing data for some variables, and prevented reliable ascertainment of ART adherence and antibiotic self-administration. Our study also was not designed to assess individual effects of NKSC component interventions on clinical outcomes.

We identified low BMI and baseline haemoglobin as patient characteristics significantly associated with death or LTFU at 12 months. Unlike other studies from SSA, women did not experience worse outcomes than men [26, 51–53]. These observations require confirmation in larger, prospective studies.

Providing comprehensive HIV-KS treatment in a rural, resource-limited setting required a novel implementation model incorporating key clinical, programmatic and psychosocial interventions. Clinically, we integrated cancer care within an effective community-based ART program, used standardized treatment protocols, task shifted care to trained clinical officers and expanded access to chemotherapy and pathology services. Programmatically, we lessened health worker workload by streamlining patient evaluation, chemotherapy administration, and clinical and laboratory monitoring through standardized protocols and forms. Lastly, we employed trained CHWs to provide psychosocial support to patients throughout HIV-KS treatment and offered transportation reimbursement to lower structural barriers to care. We postulate that the favourable outcomes observed resulted from the combined effects of these component interventions. Our model supports the concept that existing HIV services can be leveraged to provide effective cancer care for HIV-associated malignancies in SSA.

## Conclusions

We describe a successful HIV-KS treatment program characterized by excellent one-year OS comparable to resource-rich settings, infrequent adverse events and low LTFU. These results demonstrate the feasibility of expanding access to effective chemotherapy for HIV-KS in rural resource-limited settings. Our model also suggests the benefits of psychosocial interventions designed to promote retention in care and improve clinical outcomes.

We identify low baseline BMI and haemoglobin as factors associated with death or LTFU for HIV-KS in rural Malawi. Additional studies are needed to better define patient characteristics associated with HIV-KS outcomes in rural SSA. Future research should also evaluate new HIV-KS service delivery models—using established care components deployed in a manner tailored to the local context—including approaches that provide psychosocial support and ART integrated with standard-of-care chemotherapy. Our model can serve as an example for such efforts and can inform scale-up of HIV-KS treatment and care in rural SSA.

## Supplementary Material

Excellent clinical outcomes and retention in care for adults with HIV-associated Kaposi sarcoma treated with systemic chemotherapy and integrated antiretroviral therapy in rural Malawi

Excellent clinical outcomes and retention in care for adults with HIV-associated Kaposi sarcoma treated with systemic chemotherapy and integrated antiretroviral therapy in rural Malawi

Excellent clinical outcomes and retention in care for adults with HIV-associated Kaposi sarcoma treated with systemic chemotherapy and integrated antiretroviral therapy in rural Malawi

## References

[1] IARC (2012). Sub-Saharan Africa population fact sheet.

[2] Msyamboza KP, Dzamalala C, Mdokwe C, Kamiza S, Lemerani M, Dzowela T (2012). Burden of cancer in Malawi; common types, incidence and trends: national population-based cancer registry. BMC Res Notes.

[3] UNAIDS (2013). Global report: UNAIDS report on the global AIDS epidemic 2013.

[4] UNAIDS (2014). The gap report.

[5] Maskew M, MacPhail AP, Whitby D, Egger M, Fox MP (2013). Kaposi sarcoma-associated herpes virus and response to antiretroviral therapy: a prospective study of HIV-infected adults. J Acquir Immune Defic Syndr.

[6] Malope-Kgokong BI, Macphail P, Mbisa G, Ratshikhopha E, Maskew M, Stein L (2010). Kaposi's Sarcoma Associated-Herpes Virus (KSHV) seroprevalence in pregnant women in South Africa. Infect Agent Cancer.

[7] Maskew M, Macphail AP, Whitby D, Egger M, Wallis CL, Fox MP (2011). Prevalence and predictors of kaposi sarcoma herpes virus seropositivity: a cross-sectional analysis of HIV-infected adults initiating ART in Johannesburg, South Africa. Infect Agent Cancer.

[8] Adjei AA, Armah HB, Gbagbo F, Boamah I, Adu-Gyamfi C, Asare I (2008). Seroprevalence of HHV-8, CMV, and EBV among the general population in Ghana, West Africa. BMC Infect Dis.

[9] Klaskala W, Brayfield BP, Kankasa C, Bhat G, West JT, Mitchell CD (2005). Epidemiological characteristics of human herpesvirus-8 infection in a large population of antenatal women in Zambia. J Med Virol.

[10] Olweny CL, Borok M, Gudza I, Clinch J, Cheang M, Kiire CF (2005). Treatment of AIDS-associated Kaposi's sarcoma in Zimbabwe: results of a randomized quality of life focused clinical trial. Int J Cancer.

[11] Mwanda OW, Fu P, Collea R, Whalen C, Remick SC (2005). Kaposi's sarcoma in patients with and without human immunodeficiency virus infection, in a tertiary referral centre in Kenya. Ann Trop Med Parasitol.

[12] Chu KM, Mahlangeni G, Swannet S, Ford NP, Boulle A, Van Cutsem G (2010). AIDS-associated Kaposi's sarcoma is linked to advanced disease and high mortality in a primary care HIV programme in South Africa. J Int AIDS Soc.

[13] Makombe SD, Harries AD, Yu JK, Hochgesang M, Mhango E, Weigel R (2008). Outcomes of patients with Kaposi's sarcoma who start antiretroviral therapy under routine programme conditions in Malawi. Trop Doctor.

[14] Bower M, Fox P, Fife K, Gill J, Nelson M, Gazzard B (1999). Highly active anti-retroviral therapy (HAART) prolongs time to treatment failure in Kaposi's sarcoma. AIDS.

[15] Martin-Carbonero L, Barrios A, Saballs P, Sirera G, Santos J, Palacios R (2004). Pegylated liposomal doxorubicin plus highly active antiretroviral therapy versus highly active antiretroviral therapy alone in HIV patients with Kaposi's sarcoma. AIDS.

[16] Cianfrocca M, Lee S, Von Roenn J, Tulpule A, Dezube BJ, Aboulafia DM (2010). Randomized trial of paclitaxel versus pegylated liposomal doxorubicin for advanced human immunodeficiency virus-associated Kaposi sarcoma: evidence of symptom palliation from chemotherapy. Cancer.

[17] Gompels MM, Hill A, Jenkins P, Peters B, Tomlinson D, Harris JR (1992). Kaposi's sarcoma in HIV infection treated with vincristine and bleomycin. AIDS.

[18] Northfelt DW, Dezube BJ, Thommes JA, Miller BJ, Fischl MA, Friedman-Kien A (1998). Pegylated-liposomal doxorubicin versus doxorubicin, bleomycin, and vincristine in the treatment of AIDS-related Kaposi's sarcoma: results of a randomized phase III clinical trial. J Clin Oncol.

[19] Evans SR, Krown SE, Testa MA, Cooley TP, Von Roenn JH (2002). Phase II evaluation of low-dose oral etoposide for the treatment of relapsed or progressive AIDS-related Kaposi's sarcoma: an AIDS Clinical Trials Group clinical study. J Clin Oncol.

[20] Di Lorenzo G, Konstantinopoulos PA, Pantanowitz L, Di Trolio R, De Placido S, Dezube BJ (2007). Management of AIDS-related Kaposi's sarcoma. Lancet Oncol.

[21] Tulpule A, Groopman J, Saville MW, Harrington W, Friedman-Kien A, Espina BM (2002). Multicenter trial of low-dose paclitaxel in patients with advanced AIDS-related Kaposi sarcoma. Cancer.

[22] Saville MW, Lietzau J, Pluda JM, Feuerstein I, Odom J, Wilson WH (1995). Treatment of HIV-associated Kaposi's sarcoma with paclitaxel. Lancet.

[23] Stewart S, Jablonowski H, Goebel FD, Arasteh K, Spittle M, Rios A (1998). Randomized comparative trial of pegylated liposomal doxorubicin versus bleomycin and vincristine in the treatment of AIDS-related Kaposi's sarcoma. International Pegylated Liposomal Doxorubicin Study Group. J Clin Oncol.

[24] Krown SE (2011). Treatment strategies for Kaposi sarcoma in sub-Saharan Africa: challenges and opportunities. Curr Opin Oncol.

[25] Strother RM, Gregory KM, Pastakia SD, Were P, Tenge C, Busakhala N (2010). Retrospective analysis of the efficacy of gemcitabine for previously treated AIDS-associated Kaposi's sarcoma in western Kenya. Oncology.

[26] Mosam A, Shaik F, Uldrick TS, Esterhuizen T, Friedland GH, Scadden DT (2012). A randomized controlled trial of highly active antiretroviral therapy versus highly active antiretroviral therapy and chemotherapy in therapy-naive patients with HIV-associated Kaposi sarcoma in South Africa. J Acquir Immune Defic Syndr.

[27] The World Bank Group (2013). World development indicators.

[28] Neno District Executive Committee (2013). Neno district socioeconomic profile: 2013.

[29] Malawi Ministry of Health (2008). Treatment of AIDS: guidelines for the use of antiretroviral therapy in Malawi.

[30] Malawi Ministry of Health (2014). Malawi guidelines for the clinical management of HIV in children and adults. Government of Malawi, Ministry of Health.

[31] Gopal S, Krysiak R, Liomba G (2013). Building a pathology laboratory in Malawi. Lancet Oncol.

[32] Krown SE, Metroka C, Wernz JC (1989). Kaposi's sarcoma in the acquired immune deficiency syndrome: a proposal for uniform evaluation, response, and staging criteria. AIDS Clinical Trials Group Oncology Committee. J Clin Oncol.

[33] Krown SE, Testa MA, Huang J (1997). AIDS-related Kaposi's sarcoma: prospective validation of the AIDS Clinical Trials Group staging classification. AIDS Clinical Trials Group Oncology Committee. J Clin Oncol.

[34] Admon AJ, Bazile J, Makungwa H, Chingoli MA, Hirschhorn LR, Peckarsky M (2013). Assessing and improving data quality from community health workers: a successful intervention in Neno, Malawi. Public Health Action.

[35] 
Wolfe BA, Mamlin BW, Biondich PG, Fraser HS, Jazayeri D, Allen C (2006). The OpenMRS system: collaborating toward an open source EMR for developing countries. AMIA Annu Symp Proc.

[36] Division of AIDS (2009). Table for grading the severity of adult and pediatric adverse events, Version 1.0, December, 2004.

[37] Brinkhof MW, Pujades-Rodriguez M, Egger M (2009). Mortality of patients lost to follow-up in antiretroviral treatment programmes in resource-limited settings: systematic review and meta-analysis. PLoS One.

[38] Karita E, Ketter N, Price MA, Kayitenkore K, Kaleebu P, Nanvubya A (2009). CLSI-derived hematology and biochemistry reference intervals for healthy adults in eastern and southern Africa. PLoS One.

[39] Stebbing J, Sanitt A, Nelson M, Powles T, Gazzard B, Bower M (2006). A prognostic index for AIDS-associated Kaposi's sarcoma in the era of highly active antiretroviral therapy. Lancet.

[40] Borok M, Fiorillo S, Gudza I, Putnam B, Ndemera B, White IE (2010). Evaluation of plasma human herpesvirus 8 DNA as a marker of clinical outcomes during antiretroviral therapy for AIDS-related Kaposi sarcoma in Zimbabwe. Clin Infect Dis.

[41] Mlombe Y (2008). Management of HIV associated Kaposi's sarcoma in Malawi. Malawi Med J.

[42] Sissolak G, Mayaud P (2005). AIDS-related Kaposi's sarcoma: epidemiological, diagnostic, treatment and control aspects in sub-Saharan Africa. Trop Med Int Health.

[43] World Health Organization (2005). Threshold values for intervention cost-effectiveness by region.

[44] Tagar E, Sundaram M, Condliffe K, Matatiyo B, Chimbwandira F, Chilima B (2014). Multi-country analysis of treatment costs for HIV/AIDS (MATCH): facility-level ART unit cost analysis in Ethiopia, Malawi, Rwanda, South Africa and Zambia. PLoS One.

[45] Mintzer DM, Real FX, Jovino L, Krown SE (1985). Treatment of Kaposi's sarcoma and thrombocytopenia with vincristine in patients with the acquired immunodeficiency syndrome. Ann Intern Med.

[46] Mwafongo AA, Rosenberg NE, Ng'ambi W, Werner AB, Garneau WM, Gumulira J (2014). Treatment outcomes of AIDS-associated Kaposi's sarcoma under a routine antiretroviral therapy program in Lilongwe, Malawi: bleomycin/vincristine compared to vincristine monotherapy. PLoS One.

[47] Hudnall SD, Rady PL, Tyring SK, Fish JC (1998). Serologic and molecular evidence of human herpesvirus 8 activation in renal transplant recipients. J Infect Dis.

[48] Laker-Oketta MO, Wenger M, Semeere A, Castelnuovo B, Kambugu A, Lukande R (2015). Task Shifting and skin punch for the histologic diagnosis of Kaposi's sarcoma in sub-Saharan Africa: a public health solution to a public health problem. Oncology.

[49] Amerson E, Buziba N, Wabinga H, Wenger M, Bwana M, Muyindike W (2012). Diagnosing Kaposi's sarcoma (KS) in East Africa: how accurate are clinicians and pathologists?. Infect Agents Cancer.

[50] Banda LT, Parkin DM, Dzamalala CP, Liomba NG (2001). Cancer incidence in Blantyre, Malawi 1994–1998. Trop Med Int Health.

[51] Mosam A, Hurkchand HP, Cassol E, Page T, Cassol S, Bodasing U (2008). Characteristics of HIV-1-associated Kaposi's sarcoma among women and men in South Africa. Int J STD AIDS.

[52] Meditz AL, Borok M, MaWhinney S, Gudza I, Ndemera B, Gwanzura L (2007). Gender differences in AIDS-associated Kaposi sarcoma in Harare, Zimbabwe. J Acquir Immune Defic Syndr.

[53] Nasti G, Serraino D, Ridolfo A, Antinori A, Rizzardini G, Zeroli C (1999). AIDS-associated Kaposi's sarcoma is more aggressive in women: a study of 54 patients. J Acquir Immune Defic Syndr Hum Retrovirol.

