# Supplementary material for: Excellent clinical outcomes and retention in care for adults with HIV-associated Kaposi sarcoma treated with systemic chemotherapy and integrated antiretroviral therapy in rural Malawi
Source: J Int AIDS Soc. 2015 May 29;18(1):19929. doi: 10.7448/IAS.18.1.19929 (PMC4450240; doi:10.7448/IAS.18.1.19929)
Supplement: Excellent clinical outcomes and retention in care for adults with HIV-associated Kaposi sarcoma treated with systemic chemotherapy and integrated antiretroviral therapy in rural Malawi [file JIAS-18-19929-s001.pdf]

KAPOSI'S SARCOMA BODY CHART AND PATIENT EVALUATION FORM

ART #: ___ ___ ___ -- ___ ___ ___ ___ DATE: ____/____/________

Name: AGE DOB: ____ /____ /_______

NB: Complete this section ONLY at initial visit AND before Paclitaxel (Taxol)

Patient on ART (*circle*)? Yes No Indication (*circle*)? WHO III WHO IV CD4 <250 or 350

ART Start Date: ____ / ____ / ________ ARV Regimen (*circle*): 1A 2A 5A 7A

Urine Pregnancy Test (*circle*): POS NEG Other regimen (*specify*): ____________________

NB: For patients on Bleomycin+Vincristine complete at initial visit, before 10th cycle, before 20th cycle AND 4+ weeks AFTER 20th cycle.
NB: For patients on Taxol complete at initial visit, before 8th cycle AND 4+ weeks AFTER 8th cycle.

Diagnosis (*circle*): Clinical Biospy proven Date Biopsy Sample taken: _____ / _____ / ________

If Biopsy, date result reported: ____ /____ /________

Evaluation (*circle*):

Bleomycin + Vincristine Initial Visit 10 cycles 20 cycles 4+ weeks AFTER

Taxol Initial Visit 8 cycles 4+ weeks AFTER

HISTORY

Patient laying down for most of the day?	Yes	No
Patient unable to eat (due to oral lesions)?	Yes	No
Patient with difficulties walking?	Yes	No
Patient with history of oppportunistic infection since last visit or within last 6 months?	Yes	No
Patient currently having unexplained night sweats?	Yes	No
Patient with >10% weight loss from baseline or last visit?	Yes	No
Patient with diarrhea for more than 2 weeks since last visit?	Yes	No

Classification at presentation: S1 / S0 *(mark S1 if Yes to any of above)*

CLINICAL EXAMINATION:

Number of lesions (*circle*): < 25 26 - 50 >50

Find largest lesion: 1) Longest dimension (cm): ________ 2) Maximum perpendicular dimension (cm): _________

Oedema:	Yes	No
Extensive oral lesion(s):	Yes	No
Visceral lesion suspected (in lungs, GI tract, etc.):	Yes	No
Ulcerations:	Yes	No

Classification at presentation: T1 / T0 *(mark T1 if Yes to any of above)*

Bleomycin: start date: ____/ ____/ _______

Vincristine: start date: ____/ ____/ _______

Paclitaxel start date: ____/ ____/ _______

CLINICAN NAME:
