# Supplementary material for: Excellent clinical outcomes and retention in care for adults with HIV-associated Kaposi sarcoma treated with systemic chemotherapy and integrated antiretroviral therapy in rural Malawi
Source: J Int AIDS Soc. 2015 May 29;18(1):19929. doi: 10.7448/IAS.18.1.19929 (PMC4450240; doi:10.7448/IAS.18.1.19929)
Supplement: Excellent clinical outcomes and retention in care for adults with HIV-associated Kaposi sarcoma treated with systemic chemotherapy and integrated antiretroviral therapy in rural Malawi [file JIAS-18-19929-s002.pdf]

CHEMOTHERAPY ADMINISTRATION WORKSHEET

PIH/MOH Malawi :: KS Program

Patient Name:________________________________ Village/District/TA:__________________________ DOB:____________

Height:_____ KS #:_____________________ ART #:____________________________ ART Site:______________________

Cycle #	Date	Wt (kg)	BSA (m2)	Hgb	WBC	ANC	PLT	AST	ALT	Cr	Labs Safe? (Y/N)	Medication/Dose (Check One)
												☐ Vincristine 2mg IV+Bleomycin 15IU IM
												☐ Vincristine 2mg IV+Bleomycin 15IU IM
												☐ Vincristine 2mg IV+Bleomycin 15IU IM
												☐ Vincristine 2mg IV+Bleomycin 15IU IM
												☐ Vincristine 2mg IV+Bleomycin 15IU IM
												☐ Vincristine 2mg IV+Bleomycin 15IU IM
												☐ Vincristine 2mg IV+Bleomycin 15IU IM
												☐ Vincristine 2mg IV+Bleomycin 15IU IM
												☐ Vincristine 2mg IV+Bleomycin 15IU IM
												☐ Vincristine 2mg IV+Bleomycin 15IU IM

- Review FBC before administering any chemotherapy.
HOLD chemotherapy and mark 'N' in 'Labs Safe' column if:
ANC < 1000/mm^3 or **HgB < 7.0 g/dL** or **Platelets < 75K/mm^3**
- **Bleomycin** is given as a single 15 IU (1 vial) IM dose administered once weekly.
- **Vincristine** is given as a single 2mg (1 vial) IV dose administered over 5 minutes once weekly.
- **Taxol Regimen:** Taxol is given by BSA. Use the *Taxol Administration Record Sheet* to calculate Taxol dose.

Record Adverse Events Below (Hypotension, Allergic Reaction, Rash, SOB, Peripheral Neuropathy, Other)

Adverse Event:____ Date:______________

Adverse Event:____ Date:______________

Adverse Event:____ Date:______________

Adverse Event:____ Date:______________

PACLITAXEL (TAXOL) Administration Record Sheet

Name: **Village:**

Date: **Cycle #:** **VHW:**

Has the History & Physical exam sheet been completed and placed in KS register file? **Yes** **No**
Is the Consent sheet signed by patient and clinicians and placed in KS register file? **Yes** **No**

WT (kg): _____ **HT (cm):** _____ **BSA (Mosteller Formula, m2):** ____________
Mosteller formula: BSA (m^2) = $([\text{height(cm)} \times \text{weight(kg)}]/3600)^{1/2}$

Are all basic lab tests normal? ***If ANC <1000 or PLT <75,000, high SGOT/SGPT (4-5 X ULN), or jaundice, contact a physician, do NOT give TAXOL***

WBC: PLT: AST: ALT: Cr(q6wk): Jaundice: **Yes** **No**

1. Prehydration: [ ] IV NS or RL 500 ml over 3-4 hours **before** Taxol time: ____________

   [ ] Encourage additional oral fluids (water, Fanta, etc.) in the morning before Taxol

2. Premedication: [ ] Dexamethasone 20mg slow IV 60 min **before** Taxol time: ____________
   (First cycle only)
   [ ] Dexamethasone 8mg slow IV 60 min **before** Taxol time: ____________
   (All subsequent cycles)
   [ ] Promethazine 25 mg slow IV 60 min **before** Taxol time: ____________

   [ ] Cimetidine 200 mg PO 30 min **before** Taxol time: ____________

3. Immediately before giving TAXOL:

   [ ] Make sure that IV is flowing well

   [ ] Have at bedside 10ml Adrenaline 1:10,000 (1 amp of Adrenaline in 9ml of sterile water)

4. Chemotherapy: [ ] Paclitaxel 100mg/m^2 x BSA (m^2) = __ mg, divided by 6mg/ml = _____ mL of Taxol to draw up. Mix this amount in 250 mL **NORMAL SALINE only**, and give over 3 hours. To calculate rate of delivery take this total amount of solution (250mL + # mL of Taxol)

   _______ mL solution x (1/180min) x 20 drops/ml = (Drops per minute) ____________

   Starting time: ____________

   Ending time: ____________

   [ ] Check vital signs every 30 min during TAXOL administration (record TIME)

   _______ : T HR BP RR _______ : T HR BP RR

   _______ : T HR BP RR _______ : T HR BP RR

   _______ : T HR BP RR _______ : T HR BP RR

   _______ : T HR BP RR [ ] Give 10mL IV NS flush after TAXOL infusion is complete.

Carefully control possible allergic signs and IV site irritation:

- **If fever, chills, facial redness, skin eruption, dyspnea, hypotension (SBP < 90mmHG): stop IV and contact the physician immediately. Give paracetamol 500mg PO, physician to consider additional drugs (Epinephrine, etc.)**
- **If redness or burning sensation from IV site: stop IV immediately and contact the physician**

5. Post-hydration: [ ] Give IV NS or RL 500mL for 4 hours after TAXOL, and encourage oral fluids.

6. Post-medication: If patient feels nausea or is vomiting,

   [ ] Give Promethazine 25mg IV/IM, **6 hours after TAXOL infusion**

   OR

   [ ] Odansetron 8mg tab ORAL x 1, **12 hours after TAXOL infusion**

7. Prior to Discharge:

   [ ] Take out IV

   [ ] Give Ciprofloxacin 250mg, 2 tabs BDS x 5d supply (only per instructions)

   [ ] **Give Thermometer and instruct VHW/patient to take Temperature EVERY DAY, and if >38C,** instruct that patient begin Cipro and **come immediately to Neno District Hospital without delay**

   [ ] Next 3 week Appointment Date given to patient/VHW: ____________________

Treating RN: ________________________________

Treating CO: ________________________________

Treating MD: ________________________________
