# Supplementary material for: Excellent clinical outcomes and retention in care for adults with HIV-associated Kaposi sarcoma treated with systemic chemotherapy and integrated antiretroviral therapy in rural Malawi
Source: J Int AIDS Soc. 2015 May 29;18(1):19929. doi: 10.7448/IAS.18.1.19929 (PMC4450240; doi:10.7448/IAS.18.1.19929)

KAPOSI'S SARCOMA BODY CHART AND PATIENT EVALUATION FORM

BODY CHART

Draw lesions as they appear on patient (fill in lesions and cross-hatch edema)

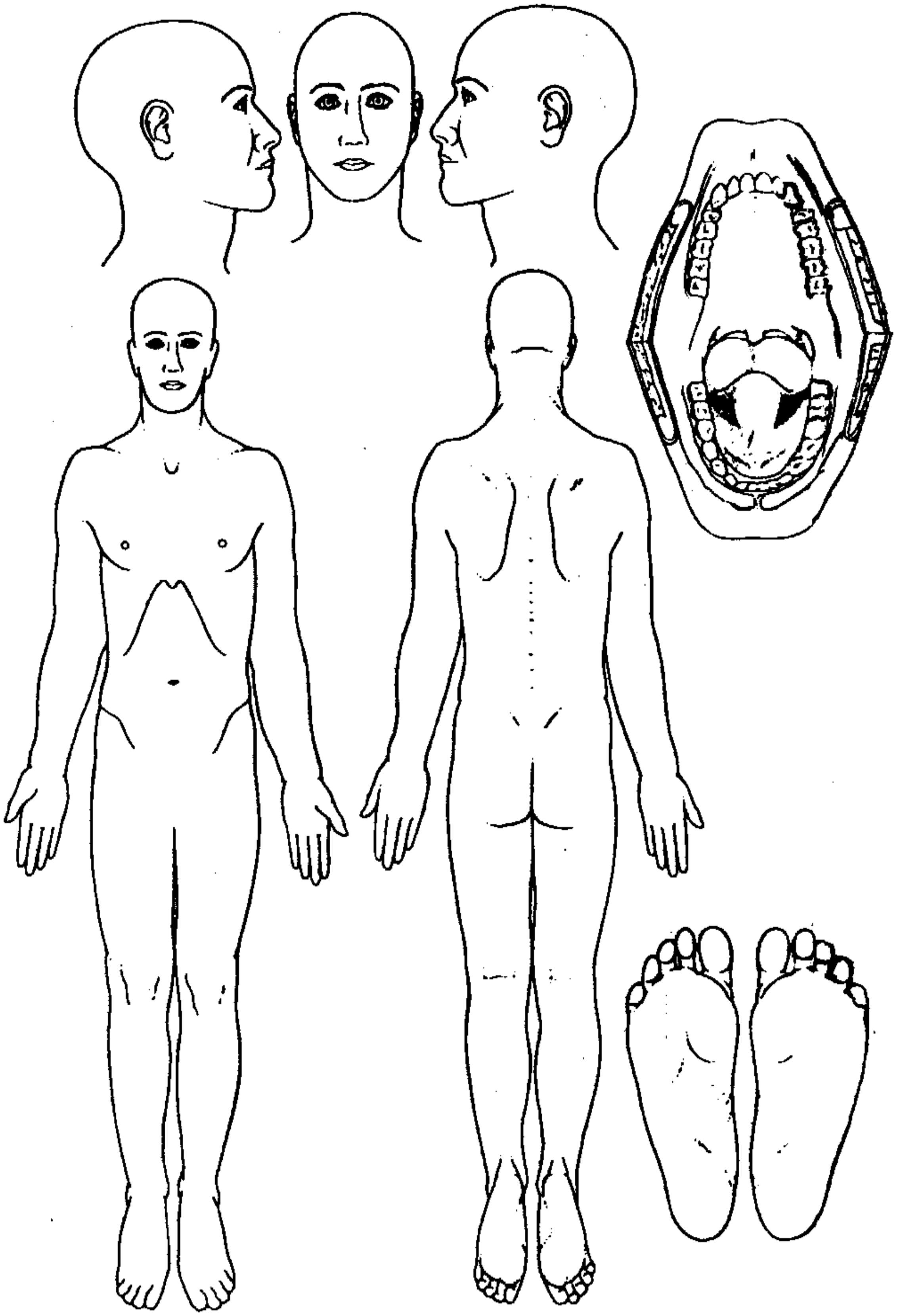

Supplement: Excellent clinical outcomes and retention in care for adults with HIV-associated Kaposi sarcoma treated with systemic chemotherapy and integrated antiretroviral therapy in rural Malawi [file JIAS-18-19929-s003.pdf]
